# In-Hospital Economic Burden of Metastatic Renal Cell Carcinoma in France in the Era of Targeted Therapies: Analysis of the French National Hospital Database from 2008 to 2013

**DOI:** 10.1371/journal.pone.0162864

**Published:** 2016-09-20

**Authors:** Rana Maroun, Franck Maunoury, Laure Benjamin, Gaëlle Nachbaur, Isabelle Durand-Zaleski

**Affiliations:** 1 GlaxoSmithKline, Marly le Roi, France; 2 Statesia, Le Mans, France; 3 URC Eco Ile-de-France, AP-HP, Paris, France; 4 ECEVE, Inserm, Paris, France; National Institute of Health, UNITED STATES

## Abstract

**Background & Objectives:**

The aim of this study was to assess the economic burden of hospitalisations for metastatic renal cell carcinoma (mRCC), to describe the patterns of prescribing expensive drugs and to explore the impact of geographic and socio-demographic factors on the use of these drugs.

**Methods:**

We performed a retrospective analysis from the French national hospitals database. Hospital stays for mRCC between 2008 and 2013 were identified by combining the 10^th^ revision of the International Classification of Diseases (ICD-10) codes for renal cell carcinoma (C64) and codes for metastases (C77 to C79). Incident cases were identified out of all hospital stays and followed till December 2013. Descriptive analyses were performed with a focus on hospital stays and patient characteristics. Costs were assessed from the perspective of the French National Health Insurance and were obtained from official diagnosis-related group tariffs for public and private hospitals.

**Results:**

A total of 15,752 adult patients were hospitalised for mRCC, corresponding to 102,613 hospital stays. Of those patients, 68% were men and the median age at first hospitalisation was 69 years [Min-Max: 18–102]. Over the study period, the hospital mortality rate reached 37%. The annual cost of managing mRCC at hospital varied between 28M€ in 2008 and 42M€ in 2012 and was mainly driven by inpatient costs. The mean annual *per capita* cost of hospital management of mRCC varied across the study period from 8,993€ (SD: €8,906) in 2008 to 10,216€ (SD: €10,527) in 2012. Analysis of the determinants of prescribing expensive drugs at hospital did not show social or territorial differences in the use of these drugs.

**Conclusion:**

This study is the first to investigate the in-hospital economic burden of mRCC in France. Results showed that in-hospital costs of managing mRCC are mainly driven by expensive drugs and inpatient costs.

## Introduction

Kidney cancer accounts for approximately 4% of all cancers in France and is the 6^th^ most common cancer in men and the 9^th^ most common cancer in women [1, 2]. In 2012, there were 11,573 new cases of kidney cancer in France: 7,781 (67%) in men and 3,792 (33%) in women [2]. Kidney cancer was responsible for 3,957 deaths in France in 2012 [2].

Renal cell carcinoma is a sub-type of kidney cancer that accounts for 85% to 92% of kidney cancer cases [3–5]. Approximately 25% to 30% of patients with renal cell carcinoma have metastases at the time of diagnosis and up to 50% of patients who undergo curative renal resection develop metastatic Renal Cell Carcinoma (mRCC) [5].

In the last decade, the prognosis of patients with mRCC has improved due to the use of targeted therapies. Indeed, overall survival has improved from 13 months to 16 months with the use of targeted therapies as compared to the use of cytokine based treatments [6]. Most of these therapeutic innovations are orally administered, which modifies the management of mRCC [7, 8]. A Danish study showed a shift in the costs of managing mRCC patients with a decrease of inpatient costs and an increase of outpatient costs [8].

Studies related to the burden of mRCC usually focus on treatment costs and even though patients still benefit from in-hospital resource consumption, the in-hospital burden of mRCC remains poorly documented. Nevertheless, for economic evaluation purposes it is important to document the in-hospital costs of mRCC regardless of their weight in the total burden of illness.

Therefore, the objectives of this study were to describe in-hospital management of mRCC, to estimate in-hospital costs, to identify in-hospital cost drivers and to study the use of expensive drugs administered at hospital.

## Materials and Methods

### Study design and data sources

A retrospective analysis was performed using data from the French national hospital database, (Programme de Médicalisation des Systèmes d’Information, PMSI), which is an exhaustive hospital discharge database that covers all hospital stays in publicly funded and private (i.e. for profit) hospitals in France. For each hospital stay, the French national hospital database includes a compilation of administrative data such as age, gender, residence code and medical data such as diagnosis (i.e. Primary Diagnosis (PD); condition that led to hospitalisation, Related Diagnosis (RD); any underlying condition which may have been related to the PD (i.e. during treatment sessions, the RD documents for which health problem the treatment is provided), and Significant Associated Diagnosis (SAD) that corresponds to complications and comorbidities which may affect the course or cost of hospitalisation, and medical procedures performed during hospitalisation. The 10^th^ revision of the International Classification of Diseases (ICD-10) is used to code data relative to diagnosis. Each hospital stay is classified in a Diagnosis Related Group (DRG) according to the PD and the medical procedures that were performed during hospitalisation. Since 2007, a unique patient identification number allows record linkage at the patient level. The DRG database is used by hospitals for reimbursement purposes and therefore only information related to the reimbursement process can be identified in the database. Since drug costs are included in the DRG tariff, drugs cannot be identified in the database. However in public hospitals, some expensive drugs usually administered at hospital and enlisted by the reimbursement authorities on a list called “expensive drug list”, are reimbursed to the hospital by the National Health Insurance in addition to the DRG-based payment. Per stay expenses related only to these so called ‘expensive drugs’ can be identified in the hospital pharmacy claims database (FICHCOMP). Since 2008, the DRG database can be linked to the hospital pharmacy claims database, which allows analysing the use of expensive drugs during hospitalisation for a given patient. This linkage is only possible for hospital stays performed in publicly funded hospitals. Indeed, expensive drugs administered in private hospitals are funded by the community based treatment funding envelope and therefore are not captured in the DRG database. In addition, oral targeted therapies in mRCC are not enlisted on the expensive drug list and cannot be identified in the DRG database.

### Hospital stays for metastatic renal cell carcinoma

We extracted all hospital stays from 2007 to 2013 with the ICD-10 codes for both renal cell carcinoma (ICD-10 code C64) and metastases (ICD-10 codes C77 to C79) as PD, RD or SAD. Of these stays for mRCC, we defined incident cases of mRCC by the absence of ICD-10 codes for mRCC or metastases in the previous year. ICD-10 codes for renal cell carcinoma and metastases are described in Table 1. Hospital stays with both renal cell carcinoma and metastases codes as SAD were not considered as incident cases.

**Table 1:** Detailed description of the ICD-codes used in this study.

ICD-10 codes	Label
***C64***	***Malignant neoplasm of kidney*, *except renal pelvis***
***C77***	***Secondary and unspecified malignant neoplasm of lymph nodes***
C77.0	Lymph nodes of head, face and neck
C77.1	Intrathoracic lymph nodes
C77.2	Intra-abdominal lymph nodes
C77.3	Axillary and upper limb lymph nodes
C77.4	Inguinal and lower limb lymph nodes
C77.5	Intrapelvic lymph nodes
C77.8	Lymph nodes of multiple regions
C77.9	Lymph node, unspecified
***C78***	***Secondary malignant neoplasm of respiratory and digestive organs***
C78.0	Secondary malignant neoplasm of lung
C78.1	Secondary malignant neoplasm of mediastinum
C78.2	Secondary malignant neoplasm of pleura
C78.3	Secondary malignant neoplasm of other and unspecified respiratory organs
C78.4	Secondary malignant neoplasm of small intestine
C78.5	Secondary malignant neoplasm of large intestine and rectum
C78.6	Secondary malignant neoplasm of retroperitoneum and peritoneum
C78.7	Secondary malignant neoplasm of liver and intrahepatic bile duct
C78.8	Secondary malignant neoplasm of other and unspecified digestive organs
***C79***	***Secondary malignant neoplasm of other and unspecified sites***
C79.0	Secondary malignant neoplasm of kidney and renal pelvis
C79.1	Secondary malignant neoplasm of bladder and other and unspecified urinary organs
C79.2	Secondary malignant neoplasm of skin
C79.3	Secondary malignant neoplasm of brain and cerebral meninges
C79.4	Secondary malignant neoplasm of other and unspecified parts of nervous system
C79.5	Secondary malignant neoplasm of bone and bone marrow
C79.6	Secondary malignant neoplasm of ovary
C79.7	Secondary malignant neoplasm of adrenal gland
C79.8	Secondary malignant neoplasm of other specified sites
C79.9	Secondary malignant neoplasm, unspecified site

**Fig 1:**
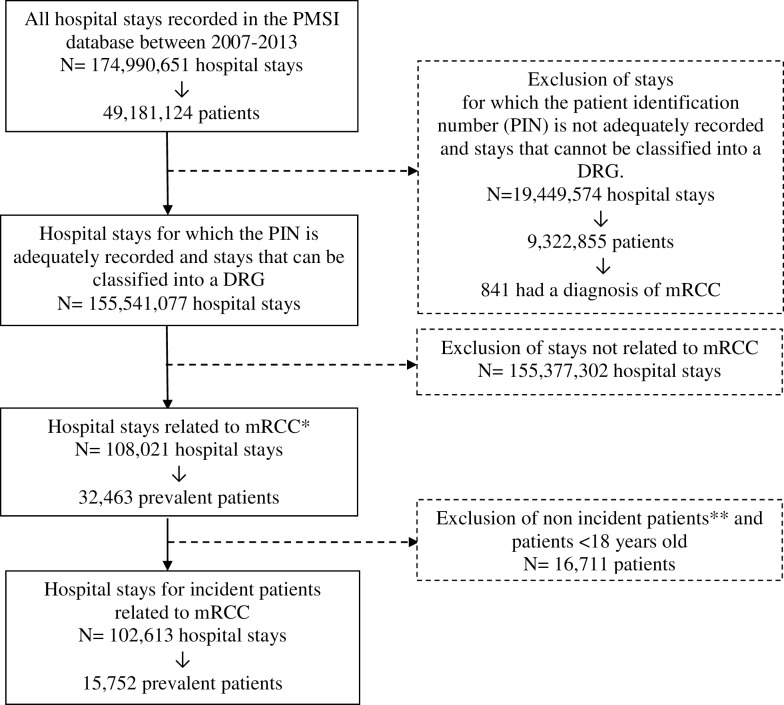
Flow chart of patients and hospital stays included in the analyses between 2008 and 2013. * Hospital stays related to mRCC are defined by the combination of two set of codes for renal cell carcinoma and for metastases (Primary Diagnosis (PD) = C64 or Related Diagnosis (RD) = C64 or Significant Associated Diagnosis (SAD) = C64) and ((PD = C77 or or C78 or C79) or (RD = C77 or C78 or C79) or (SAD = C77 or C78 or C79)). ** Patients for whom a hospitalisation related to mRCC or a code of metastases were identified in the previous year, or where both metastases and renal cell carcinoma were coded as an SAD (SAD = C64 + SAD = (C77 or C78 or C79)).

Hospital stays for which the patient identification number was not adequately recorded as well as hospital stays that could not be classified into a DRG were excluded. In addition hospital stays for non incident cases were excluded (Fig 1). Outpatient hospitalisations are defined as hospital stays without an overnight stay and during which patients are delivered polyvalent and intensive care. Inpatient hospitalisations are hospital stays with overnight stay.

### Assessing the number of patients and describing their characteristics

The number of hospitalised patients for mRCC was obtained by linking all hospital stays to the unique patient identification number. Patients under 18 years were excluded. Only deaths occurring during a hospital stay are recorded within the DRG database (cause of death is not recorded) and were therefore described as hospital mortality rates in our analysis.

Hospital mortality rates were calculated as the number of deaths for mRCC divided by the total number of hospitalised persons presenting with mRCC multiplied by 100 [9].

### Patterns of prescribing expensive drugs

Prescription of expensive drugs was described and presented by treatment frequency, percentage of hospital stays leading to a prescription of an expensive drug, number of patients receiving at least one expensive drug, patient gender, rurality of the residence of the patient and social deprivation index related to the town of residence of the patient. The social deprivation index used in this analysis was the Fdep2008 [10, 11]. This index was calculated for each town based on four variables: unemployment rate, median household income, percentage of high school graduates in the adult population and percentage of blue-collar workers in the active population and divided into five categories (1st quintile: most privileged; 5th quintile: most deprived).

### Estimating hospital costs

Costs were estimated from the perspective of the French National Health Insurance. Costs were calculated using the published DRG tariffs for 2015. DRG tariffs cover treatments (except expensive drugs), medical procedures, nursing and physician fees. DRG tariffs for private hospitals do not include physician fees which are paid in addition to the DRG. In addition for private hospitals, costs of expensive drugs and radiotherapy could not be taken into consideration because these data are not available. For private hospitals, physician fees, radiotherapy sessions and expensive drugs are funded by the ambulatory funding envelope and therefore are not captured in the DRG database. When applicable, additional costs such as costs of hospitalisation in an intensive care unit, costs of radiotherapy and dialysis were added to DRG tariffs. Costs are presented as total cost over the period, total cost per year, mean cost per patient per year and per type of hospital stay, mean cost per patient per year of inclusion and per type of hospital stay. All costs are presented in Euros 2015.

### Statistical analyses

Statistical analyses were performed using SAS 9.3. Analyses of hospital stays and patients were descriptive. Categorical variables were described as percentages, and continuous variables were summarized as means (SD) or medians [Min-Max]. Statistics for hospital stays are presented as number of discharges and mean (SD) length of stay. Statistics for patients are presented as number of new cases, as percentage of in-hospital deaths, percentage of men, median age [Min-Max], most frequent localization of metastases (metastases occurring in more than 15% of cases). Descriptive analyses were performed on treatment patterns, bivariate and multivariate logistic regression analyses were performed to analyze the impact of socio-demographic characteristics on the use of expensive hospital drugs. The dependent variable was the use of expensive drugs in patients with mRCC and explanatory variables corresponded to age, gender, rurality and social deprivation index.

### Ethics

Ethics committee approval was not required for this study, authorization to access and analyze the DRG database was granted by the French data protection authority (Commission Nationale de l’Informatique et des Libertés, CNIL) (CNIL authorization: N°1846495). Anonymized data were provided by the French Agency responsible for collecting information on hospitalisations (Agence Technique de l'Information sur l'Hospitalisation, ATIH) under the agreement number 2015-100011-30-85.

## Results

### Characteristics of patients hospitalised for mRCC

**Fig 2:**
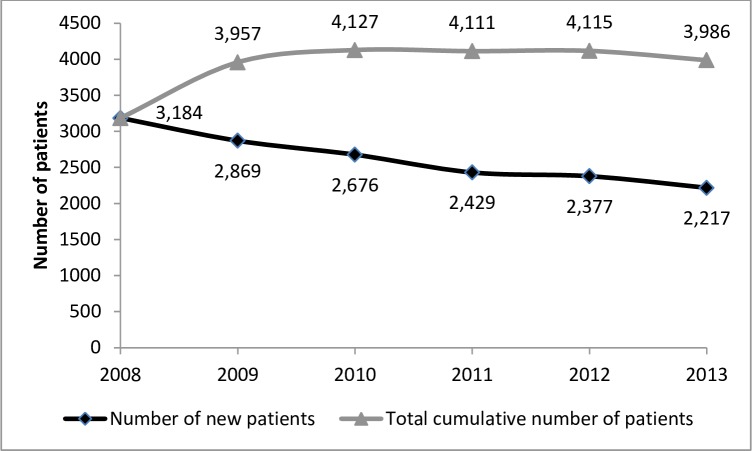
Evolution of the number of patients between 2008 and 2013.

Between 2008 and 2013, 15,752 adult patients were hospitalised for mRCC in France (Fig 1). The number of patients who had a first hospitalisation related to mRCC slightly decreased between 2008 (n = 3,184) and 2013 (n = 2,217). However, the cumulative number of patients remained stable across the study period (Fig 2).

Most of the 15,752 patients were men (68%) and the median age at first hospital stay was 69 years [Min-Max: 18–102]. 59% of these patients had multiple sites of metastases. The most frequent sites of metastases were lung (n = 8,446 patients), bone & bone marrow (n = 6,313 patients), liver (n = 4,382 patients) and brain (n = 2,456 patients). Of the 15,752 patients, 406 (3%) had at least one other cancer. Among these 406 patients, the most frequent sites of cancer were the digestive tract 23% (n = 93), the respiratory tract 23% (n = 93), and the urinary tract 10% (n = 41) (Table 2). Over the study period 5,801 patients died at hospital corresponding to a hospital mortality rate of 37%.

**Table 2:** Patient's characteristics.

**Age; years**	
Mean (SD)	68 (13)
Median [Min-Max	69 [18–102]
**Gender; n (%)**	
Men	10,654 (68%)
Women	5,098 (32%)
**Number of sites of metastases; n (%)**	
1	6,428 (41%)
2	3,993 (25%)
3	2,552 (16%)
≥4	2,779 (18%)
Most frequent localization of metastases; n (%)*	
Lung	8,446 (54%)
Bone & bone marrow	6,313 (40%)
Liver	4,382 (28%)
Brain	4,382 (28%)
**Concomitant cancers; n (%)**	**406 (3%)**
Digestive tract **	93 (23%)
Respiratory tract**	93 (23%)
Urinary tract **	41 (10%)

* Percentages could exceed 100% because individual patients could have multiple sites of metastases.

**Proportion of the 406 patients who had another concomitant cancer.

### Description of hospital stays

Over the study period (2008–2013), there were 102,613 hospital stays related to mRCC of which 80% took place in publicly funded hospitals. Of these hospital stays 65,485 (64%) were outpatient admissions, of which chemotherapy and radiotherapy sessions accounted for 82% (n = 53,826). The principal reasons for inpatient hospitalisations were medical treatment of cancer (56% of stays) and chemotherapy sessions (6%). Surgery and palliative care represented 22% and 16% respectively of all inpatient hospitalisations. For inpatient hospitalisations, the overall mean length of stay was 12 days (SD: 14) and the median length of stay 8 days [Min-Max: 1–369]. When the analysis was restricted to palliative care, the mean length of stay was 18 days (SD: 17) and the median length 13 days [Min-Max: 1–251].

### Patterns of prescribing expensive drugs

Expensive drugs were prescribed in 26% (N = 21,136) of admissions in publicly funded hospitals, nearly always during an outpatient admission. Among the 12,542 patients hospitalised in a publicly funded hospital, 16% (N = 1,972) received at least one prescription of expensive drugs. Patients receiving expensive drugs tended to be younger (median age: 64 years [Min-Max: 18–93]) than those who did not receive expensive drugs (median age: 70 years [Min-Max: 18–102]) (Table 3). Temsirolimus was prescribed in 68% of cases and bevacizumab in 30% of cases other expensive drugs were prescribed in 2% of cases.

**Table 3:** Distribution of patients according to socio-demographic variables and prescription of expensive drugs.

	Patients with prescription of expensive drugs* N = 1,972	Patients without prescription of expensive drugs* N = 10,570
**Age (years) median [Min-Max]**	64 [18–93]	70 [18–102]
**Gender (% men)**	1,388 (70%)	7,044 (67%)
Rurality**		
Rural	899 (46%)	4,997 (47%)
Semi-Rural	373 (19%)	1,885 (18%)
Semi-Urban	442 (23%)	2,370 (22%)
Urban	239 (12%)	1,196 (11%)
Missing	19 (1%)	122 (1%)
**Social deprivation Index**		
1^st^ quintile	483 (25%)	2,542 (24%)
2sd quintile	401 (21%)	2,045 (20%)
3d quintile	379 (19%)	2,020 (19%)
4^th^ quintile	386 (20%)	2,253 (22%)
5^th^ quintile	304 (16%)	1,588 (15%)

*These analyses were conducted only in patients admitted to a publicly funded hospital.

**Patients for whom rurality was not documented were excluded from the analysis of the social deprivation index. The 1^st^ quintile represents the most privileged patients and the 5^th^ quintile represents the most deprived patients.

Multivariate analysis of expensive drugs utilisation in hospital did not show any significant difference between rural and urban areas (OR = 0.976) IC95% [0.751–1.269] or between the most deprived patients and the most privileged ones (OR = 1.123) IC95% [0.868–1.451]. However, being an elderly patient was associated with a lesser use of expensive drugs OR = 0.984 IC95% [0.979–0.990] (Table 4).

**Table 4:** Multivariate logistic analysis of the impact of socio-demographic variables on the use of expensive drugs.

	OR*	IC
**Age**	0.984	0.979–0.990
**Gender**		
Women	Reference	Reference
Men	1.044	0.885–1.233
Rurality**		
Urban	Reference	Reference
Semi-urban	0.973	0.739–1.282
Semi-rural	1.014	0.763–1.347
Rural	0.976	0.751–1.269
Social deprivation index**		
1^st^ quintile	Reference	Reference
2sd quintile	1.222	0.975–1.533
3^rd^ quintile	1.016	0.792–1.302
4^th^ quintile	0.976	0.764–1.248
5^th^ quintile	1.123	0.868–1.451

*These analyses were conducted only in patients admitted to publicly funded hospitals.

**Patients for whom rurality or social deprivation index were missing were excluded from the analysis. The 1^st^ quintile represents the most privileged patients and the 5^th^ quintile represents the most deprived patients.

### Costs of managing mRCC at hospital

The mean annual costs per patient for outpatient hospitalisations and inpatient hospitalisations were respectively 7,413€ (SD: 15,679) and 12,259€ (SD: 11,348). The mean annual cost of hospital management of mRCC varied across the study period from 8,993€ per patient (SD: €8,906) in 2008 to 10,216€ per patient (SD: €10,527) in 2012. Heterogeneity was observed between publicly funded hospitals on the one hand and private hospitals on the other. Between 2008 and 2013, the mean cost of hospital care for mRCC varied between 4,777€ per patient (SD: €3,802) in 2008 and 5,371€ (SD: €5,322) per patient in 2012 in private hospitals vs. 9,899€ per patient (SD: €9,477) in 2008 and 11,068€ per patient (SD: 11,054) in 2012 in publicly funded hospitals. The distribution of the mean annual cost per patient according to the type of hospital stay is presented in Table 5. The mean annual cost of patients in palliative care was 9,659€ (SD: €8,443) per patient and the mean cost of stays where the patient dies in hospital was 7,230€ (SD: €6,203) per patient.

**Table 5:** Distribution of the mean cost per patient and total annual cost according to the type of hospital stay.

Year	Number of patients	Number of patients who received an expensive drug	Mean cost per patient (€) ± (SD)					Total annual cost from the payer perspective (€)					
			Outpatient Public	Outpatient private	Inpatient public	Inpatient private	Expensive drugs*	Outpatient private	Outpatient public	Inpatient public	Inpatient private	Expensive drugs*	Total
**2008**	3,184	376	2,987 (3,157)	1,884 (2,011)	8,756 (7,686)	4,892 (3,672)	8,337 (8,183)	431,463	2,676,088	18,194,697	4,197,700	3,134,594	28,634,542
**2009**	3,957	584	2,986 (3,506)	1,957 (2,484)	9,178 (7,823)	5,014 (4,211)	9,256 (10,558)	704,556	3,792,604	23,834,942	5,114,155	5,404,765	38,851,022
**2010**	4,127	502	2,961 (3,433)	2,279 (2,771)	9,026 (8,161)	5,042 (4,915)	9,689 (9,635)	804,530	3,852,129	25,625,279	5,559,184	4,864,023	40,705,145
**2011**	4,111	456	2,884 (3,121)	2,166 (2,425)	9,316 (8,080)	5,503 (4,626)	9,306 (9,860)	797,143	3,754,568	26,188,085	5,740,051	4,243,536	40,723,383
**2012**	4,115	392	2,752 (3,085)	2,073 (2,482)	9,799 (9,319)	5,500 (5,286)	9,797 (10,107)	739,991	3,583,222	28,104,979	5,769,442	3,840,597	42,038,231
**2013**	3,986	317	2,630 (3,026)	1,641 (2,107)	9,854 (9,411)	5,353 (4,034)	9,834 (9,834)	421,768	3,179,439	27,671,161	5,299,833	3,117,318	39,689,519

*Only for publicly funded hospitals

For incident patients, the mean annual costs per patient by type of hospital stay in 2008 were: 7,695€ (SD: €6,920) for inpatient hospitalisations and 2,114€ (SD: €2,397) for outpatient hospitalisations. For these patients, the mean cost of expensive drugs was 5,971€ per patient (SD: €6,082). In 2013, the mean costs per incident patient by type of hospital stay were: 8,935€ (SD: €8,801) for inpatient hospitalisations and 1,934€ (SD: €2,026) for outpatient hospitalisations, the mean cost of expensive drugs being 6,277€ per patient (SD: €7,269). The distribution of the mean annual cost per patient according to the year and to the type of hospital stay is presented in Table 6.

**Table 6:** Distribution of the annual mean cost per incident patient according to the type of hospital stay and the year of inclusion in the cohort.

Year of inclusion	Number of incident patients	Number of incident patients who received an expensive drug	Mean annual cost per patient (€) ± (SD)				
			Outpatient private	Outpatient public	Inpatient public	Inpatient private	Expensive drugs*
**2008**	3,184	561	1,317 (1,692)	2,202 (2,488)	7,876 (7,213)	3,799 (3,537)	5,971 (6,082)
**2009**	2,869	456	1,133 (1,401)	1,901 (2,142)	8,182 (6,740)	4,149 (3,952)	5,231 (5,519)
**2010**	2,676	346	1,314 (1,560)	1,862 (2,047)	8,382 (7,508)	4,649 (4,704)	5,612 (5,381)
**2011**	2,429	268	1,393 (1,649)	1,857 (2,064)	8,822 (7,480)	4,981 (4,587)	5,392 (5,752)
**2012**	2,377	215	1,393 (1,981)	1,828 (1,986)	9,349 (8,122)	5,152 (5,614)	5,990 (6,716)
**2013**	2,217	126	1,422 (1,968)	2,019 (2,007)	9,682 (9,510)	5,390 (3,818)	6,277 (7,269)

*Only for publicly funded hospitals

Over the entire study period, the total direct hospital costs for the cohort (N = 15,752 patients) represented 230,641,841€, of which the cost of expensive drugs accounted for 11% (24,604,833€). The distribution of annual costs according to the type of hospital stay is presented in Table 5.

## Discussion

We estimated the in-hospital economic burden of metastatic renal cell carcinoma from the perspective of the French National Health Insurance through a retrospective analysis of the French DRG database (PMSI database).

Our results indicate that 15,752 incident patients were hospitalised for mRCC between 2008 and 2013. 68% of patients included in the analysis were male and the median age at first hospitalisation was 69 years. These results are consistent with epidemiological data available in the literature [3, 12].

The analysis of the determinants of prescribing expensive drugs in publicly funded hospitals did not reveal social and territorial differences in the use of these drugs. However, older age seems to be associated with lesser use of expensive drugs (OR = 0.984 IC95% [0.979–0.990]). Given the limited clinical data available in the PMSI database, these results should be interpreted with caution. Indeed, the model did not take into consideration certain potential confounding variables such as comorbidities and time since diagnosis. With this respect, our results differ from the results of the TERRITOIRE study, which has evaluated access of patients with lung cancer to expensive drugs in France as a function of geographical and socio-demographic factors, and which demonstrated some lack of social equity in access to expensive drugs [13]. The differences between our results and those of the TERRITOIRE study may be explained by the fact that we used a different version of the deprivation index; that the impact of social equity in using expensive drugs might vary according to the type of disease and other confounding variables such as comorbidities. We observed a sustain decrease in the number of patients who received at least one expensive drug by year of inclusion. This decrease might be explained by the increasing use of oral anticancer treatments [14]. Aggregate market research data showed a 2 fold increase in oral targeted therapies indicated in mRCC between 2008 and 2013 [15]. However, we do not dispose of the adequate data to confirm that the decrease in the use of in-hospital expensive drugs is due to an increasing use of oral targeted therapies. This study estimated the in-hospital economic burden of mRCC in France over a 6 year period. The mean annual costs per patient for outpatient hospitalisations and inpatient hospitalisations were respectively 7,413€ and 12,259€. In a Danish study, outpatient visits and inpatient hospitalisations were estimated to cost respectively 14,308€ and 11,899€ per patient per year. The Mean annual cost per patient for outpatient hospitalisations in our study was considerably lower than the estimated costs in the Danish study [8]. These differences might be explained by differences in health resource utilisation and related DRG tariffs between the two countries. Our findings also showed that the main drivers of the overall cost of hospital management were inpatient hospitalisations and expensive drugs. The mean annual cost per patient by year of inclusion and type of hospital stay as well as the mean annual cost per patient by type of hospital stay varied slightly. Since we used a one year tariff and the mean number of stays by year of inclusion was stable, these differences may be explained by variability in the structure of healthcare resource utilisation over time.

However, this study has some limitations which should be taken into consideration. Physician fees for the private sector are not taken into consideration in the DRG tariffs and sessions of radiotherapy performed in the private sector, as well as expensive drugs delivered during private hospitalisation are not captured by the DRG database. Therefore, the in-hospital burden for private hospitals is underestimated in our study. Nonetheless, this limitation is restricted to the quantification of the economic burden of mRCC and does not have an impact on the description of the medical burden. These limitations could partly explain the heterogeneity observed between publicly funded and private settings.

Furthermore, this study does not estimate the global burden of mRCC in France but focuses on the in-hospital economic burden. Studies related to the burden of mRCC usually focus on treatment costs and even though patients still benefit from in-hospital resource consumption, the in-hospital burden of mRCC is not well documented. To estimate the in-hospital burden of mRCC, we used the national DRG database which is considered as a reliable and exhaustive source to collect information on hospitalisations. However, the DRG database does not capture healthcare costs generated in the community. Indeed, in France resource consumption in community settings are funded separately and cannot be identified in the DRG database and access to these data is restricted.

Even though our study doesn’t take into consideration the economic burden of mRCC in the community, it highlights the in-hospital economic burden of mRCC and constitutes a first step in evaluating the total burden of mRCC. Information regarding the economic burden of mRCC in France is scarce and this study contributes in documenting the in-hospital economic burden of mRCC which needs to be assessed regardless of its weight in the total burden.

Another limit is that this study does not allow the real evolution of costs in the management of mRCC at hospital to be captured, since we valued the resource use by using standard one-year 2015 DRG tariffs for each year of the study.

Furthermore, we could only estimate mortality from in-hospital deaths, which do not reflect the global mortality in patients with mRCC.

Despite these limitations, the present study has estimated the cost of hospitalisation for mRCC in France for the first time and provides a basis for future economic evaluations of new treatments for mRCC.

## Conclusions

The present study evaluated the cost of hospitalisations for mRCC and the burden of mRCC at hospital. The cost of hospitalisations is only a part of the total cost of management of mRCC and further research is needed in order to capture the community healthcare costs of mRCC and thus provide a full description of the economic and medical burden of mRCC in order to be used for economic evaluation of new therapies.
